# Red Tape and Efficiency

**Published:** 1917-09

**Authors:** 


### Red Tape and Efficiency.

As many physicians will enter government service, let us say a few words about the above topics. Any of us could take a position as president or manager or superintendent of a large business. On the first day of our new duties, we would discover a great many unnecessary, complicated and time and money-consuming methods. The elimination of these methods and the substitution of simple and practical ones would at once occur to us. But, at the end of a few months, the business would probably be in the same condition as a patient with pericarditis or appendiceal abscess if treated by the person whose place we took. Red tape means the business methods of government. It has been developed very gradually by men of long experience, mainly of ability and conscientiously devoted to their profession. It is ultimately intended to protect the stock holders who are the people. An army surgeon who, as a young man, was a clerk in an administrative department of a railroad, declares that the business forms and system of the Surgeon General's office and of the entire medical corps are far simpler and far more efficient than those of any railroad in the country.

As to efficiency, remember that it depends largely on learning the real nature of a new line of work. Don't be like the type-writer manufacturers who advertise a medical key-board on the strength of drachm, ounce and scruple signs. On the other hand, don't be too critical if you encounter survivals of the past encumbering 20th century methods. Even the scruple has not entirely disappeared from medical writings.

